# Gaussian Process Regressions for Inverse Problems and Parameter Searches in Models of Ventricular Mechanics

**DOI:** 10.3389/fphys.2018.01002

**Published:** 2018-08-14

**Authors:** Paolo Di Achille, Ahmed Harouni, Svyatoslav Khamzin, Olga Solovyova, John J. Rice, Viatcheslav Gurev

**Affiliations:** ^1^Healthcare and Life Sciences Research, IBM T.J. Watson Research Center, Yorktown Heights, NY, United States; ^2^IBM Research Almaden, San Jose, CA, United States; ^3^Ural Federal University, Yekaterinburg, Russia; ^4^Institute of Immunology and Physiology, Ural Branch of the Russian Academy of Sciences (UB RAS), Yekaterinburg, Russia

**Keywords:** LV mechanics, FEM, infarct model, unloaded configuration, kriging, inverse optimization, statistical learning

## Abstract

Patient specific models of ventricular mechanics require the optimization of their many parameters under the uncertainties associated with imaging of cardiac function. We present a strategy to reduce the complexity of parametric searches for 3-D FE models of left ventricular contraction. The study employs automatic image segmentation and analysis of an image database to gain geometric features for several classes of patients. Statistical distributions of geometric parameters are then used to design parametric studies investigating the effects of: (1) passive material properties during ventricular filling, and (2) infarct geometry on ventricular contraction in patients after a heart attack. Gaussian Process regression is used in both cases to build statistical models trained on the results of biophysical FEM simulations. The first statistical model estimates unloaded configurations based on either the intraventricular pressure or the end-diastolic fiber strain. The technique provides an alternative to the standard fixed-point iteration algorithm, which is more computationally expensive when used to unload more than 10 ventricles. The second statistical model captures the effects of varying infarct geometries on cardiac output. For training, we designed high resolution models of non-transmural infarcts including refinements of the border zone around the lesion. This study is a first effort in developing a platform combining HPC models and machine learning to investigate cardiac function in heart failure patients with the goal of assisting clinical diagnostics.

## 1. Introduction

Multi-scale models of cardiac mechanics, although are promising (e.g., Kerckhoffs et al., 2007; Nordsletten et al., 2011; Gurev et al., 2015; Land et al., 2017), have found limited applications for diagnosis and treatment. To reach the levels of accuracy needed to assist clinical decisions, models need to overcome major complications related to accessing clinical data, constraining unknown parameters, and coping with computational complexity. Some of the uncertainties associated to patient-specific cardiac models can be partially addressed with increased public access to large clinical datasets (Fonseca et al., 2011) and to high performance computing resources (Towns et al., 2014). Sophisticated finite element (FE) biomechanical simulations can be combined with machine learning techniques to translate parametric studies into efficient statistical models of virtual patient populations. Once an upfront computational cost is paid for training, the coupled effects of varying model parameters can be explored almost in real time, facilitating the solution of the optimization and inverse estimation problems that are required to personalize models for specific patients.

This paper discusses statistical models based on a machine learning technique called Gaussian Process (GP) regression, also known as kriging (Rasmussen and Williams, 2006). After training a “surrogate” of the more expensive FE models, GP regression can be used to assist optimization algorithms, even in complex cases where objective functionals cannot be easily differentiated (Booker et al., 1999; Abramson et al., 2009). More recently, GP regression has also been used in cardiovascular modeling, where it has found application in both fluid and solid mechanics (Marsden et al., 2008; Sankaran and Marsden, 2011; Pérez et al., 2016).

Recent developments in medical imaging techniques have opened new opportunities for cardiac modeling to augment image-based biomarkers from CT, MRI, and ultrasound scans (Lamata et al., 2014). As accuracy and availability of imaging modalities continues to improve, there is a growing need for novel strategies that exploit the capabilities of multi-scale models to enhance diagnostic tools. We present a systematic analysis of the Sunnybrook Cardiac MRI database, a public collection of cine-MRIs (Radau et al., 2009). Statistics gathered from the database were used to design two parametric studies investigating the passive behavior of the myocardium upon inflation and the effects of infarct on cardiac performance.

In the first parametric study, we developed a novel strategy to estimate the unloaded configuration (needed to initialize both passive and active FEM simulations) given either the end-diastolic intraventricular pressure, or the end-diastolic fiber strain. The new method relies on solving multiple forward problems to train a regression model from which unloaded configurations can be inferred for ventricles with arbitrary shapes. Despite such a problem could be alternatively solved with the fixed point iteration method (Sellier, 2011; Genet et al., 2015), our approach has some advantages. Specifically, our method can be easily applied in situations where the intraventricular pressure is not directly known (but could be inferred, for example, from the fiber strain), or where the unloaded geometry is one of the unknown parameters of an optimization problem.

The second example integrates machine learning and multi-scale modeling in a systematic parametric study investigating the effects of infarct on simulated cardiac performance. Location, size, and transmural depth of the infarct were chosen as input variables of a GP regression model predicting changes in simulated stroke volume due to the scar. This work exploited the capabilities of our in-house solver and an automatized workflow to run 40 simulations of infarct with varying shapes and locations. After training on results of FE simulations, the GP regression model provides a useful representation for the analysis of complex effects. Non-transmural infarcts were simulated with a high numerical accuracy.

## 2. Methods

### 2.1. Cine-MRI segmentations and parameterization via idealized models

Publicly available imaging datasets from the Sunnybrook Cardiac MRI database (Radau et al., 2009) were systematically processed to establish boundaries and proper feature distribution for parametric exploration. The Sunnybrook database gathered 45 cine-MRI scans collected from healthy subjects (*N, n* = 9), patients with ventricular hypertrophy (*HYP, n* = 12), and patients affected by heart failure both in presence and absence of myocardial infarction (*HF-I, n* = 12 and *HF-NI, n* = 12, respectively). For each scan, we considered only the short axis stack series, which provided ~10–15 axial slices per left ventricle (LV) and 20 frames per cardiac cycle. Average voxel sizes were (1.36 ± 0.057 mm) × (1.36 ± 0.057 mm) × (8.8 ± 1.0 mm) in the left-right, anterior-posterior, and apical-basal directions, respectively.

**Figure 1:**
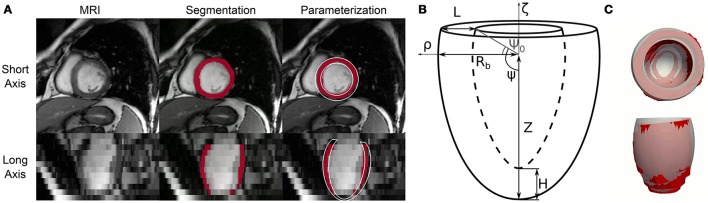
Automatic processing of cine-MRI images from the Sunnybrook Cardiac MRI database and fitting of idealized geometric model. **(A)** Complete processing of a representative short axis view frame from patient I-01 in the database. This Cine-MRI modality showed sufficient in-plane resolution, but significantly lower detail in the long axis view (e.g., compare first and second row of the first column). An atlas-based image processing algorithm was employed to extract LV boundaries for each patient. Segmented pixels are shown marked in red in the second and third column. Finally, an idealized 6-parameter model of LV geometry was fitted to the segmentation results, partially correcting for the artifacts introduced by the low resolution in the long axis (see model cross-section rendered in white in the third column). **(B)** Geometric meaning of idealized LV model parameters. Radial and axial coordinates are indicatd by ρ and ζ, respectively. *R*_*b*_ = outer radius at base, *L* = wall thickness at base, *Z* = distance from center of the ventricle to apex of outer wall, *H* = wall thickness at apex, Ψ_0_ = truncation angle. Not shown is the *e* parameter, which governs the curvature of the LV external and internal walls. More details on analytical expressions of the LV geometric profile are provided in the text. **(C)** Top and lateral 3-D views of overlapped segmentation (rendered as a red surface) and best-fit idealized model (rendered as a gray transparent overlay) for a representative case (I-01 at beginning of diastole).

An in-house multi-atlas image processing technique (Xie et al., 2015) was used to co-register the axial slices of each dataset and then segment the LV boundaries. The first 2 columns of Figure 1A show the procedure applied to a representative 3-D image from the database. Outputs were labeled voxels marking the LV blood pool (shown in white semi-transparent overlay) and the ventricular wall (shown in red). The low resolution in the apical-basis direction typical of cine-MRI short axis views introduced segmentation artifacts that prevented direct use in FEM models. We therefore performed a further parameterization step (see third column) to approximate LV geometries as truncated prolate spheroids, as initially proposed by Streeter and Hanna (1973) and more recently revisited by Pravdin et al. (2014). According to such a scheme, the endocardial and epicardial profiles of an idealized axisymmetric LV were described by the following relations

(1) $$\begin{array}{l} \rho_{epi}=R_{b}\left[e\cos\psi +\left(1-e\right)\left(1-\sin\psi \right)\right]\\ \zeta_{epi}=Z\left(1-\sin\psi \right)\\ \rho_{end}=\left(R_{b}-L\right)\left[e\cos\psi +\left(1-e\right)\left(1-\sin\psi \right)\right]\\ \zeta_{end}=\left(Z-H\right)\left(1-\sin\psi \right)+H \end{array}$$

linking the radial (ρ) and axial (ζ) coordinates of the epicardial and endocardial boundaries to the angle variable ψ ∈ [ψ_0_, π/2]. In the equations above, the idealized geometry is defined by 6 parameters: the outer radius at base, *R*_*b*_; the length of the longitudinal semi-axis of the outer spheroid, *Z*; the ventricular wall thicknesses at base and apex, *L* and *H*, respectively; the sphericity/conicity of the spheroid, *e* ∈ [0, 1]; and, finally, the truncation angle, ψ_0_. Figure 1B shows a schematic of an idealized LV annotated with geometric descriptions of the parameters.

In order to describe the segmentation results in terms of the idealized models described above, we implemented an *ad hoc* optimization procedure to find sets of parameters **ξ** = {*R*_*b*_, *Z, L, H, e*, Ψ_0_} that would best match the MRI segmentations (*I*_MR_). Each iteration involved first generating a binary 3-D image *I*_**ξ**_ marking the LV volume defined by **ξ**, and then evaluating an objective function *J* defined as

(2) $$\begin{array}{l} J\left(I_{\xi },I_{\text{MR}}\right)=1-\frac{1}{2}\left(\frac{C_{\xi }\;\cap \;C_{\text{MR}}}{C_{\xi }\;\cup \;C_{\text{MR}}}+\frac{W_{\xi }\;\cap \;W_{\text{MR}}}{W_{\xi }\;\cup \;W_{\text{MR}}}\right), \end{array}$$

where *C*_**ξ**_ and *C*_MR_ indicate the ventricular cavity regions in the idealized and MR segmentation images, respectively; and *W*_**ξ**_ and *W*_MR_ similarly indicate corresponding ventricular wall volumes. In other words, *J* ∈ [0, 1] provides a measure of similarity between a “synthetic” segmentation *I*_**ξ**_ generated for any given ξ and the actual MRI processing results *I*_MR_. The “Nelder-Mead” algorithm available in SciPy was used to carry out the optimization up to convergence for every image dataset included in the database.

The relations in (1) do not include any parameters accounting for the rigid translation and rotations that LVs normally experience during a cardiac cycle. To overcome such limitation and to improve fitting results, each objective function evaluation was preceded by a rigid transformation step aimed at aligning the idealized model to the target segmented geometry. Specifically, we first estimated the main longitudinal axis of the segmented ventricle as the best-fit direction aligning the centers of gravity of the LV segmented axial slices. We then rigidly transformed the idealized models to let the longitudinal axes and the centers of gravity of the two geometries coincide. Figure 1C shows overlapped optimization results and corresponding MRI segmentation for a representative cine-MRI frame after rigid motion correction.

### 2.2. Passive material properties

To assess whether the inverse esimation method presented in this work would generalize to describe other constitutive behaviors (e.g., from future experiments on animal and human tissues, or from novel modeling developments), we considered 3 sets of material parameters (and related functional formulations) from the literature that describe experimental findings on canine, swine, and human ventricle biomechanics. Usyk et al. (2000) fitted a Fung-type orthotropic strain energy function to experiments on canine models

(3) $$\begin{array}{l} W_{U}=\frac{C}{2}\left(\text{exp}\left(Q\right)-1\right),\;Q=b_{\text{ff}}E_{\text{ff}}^{2}+b_{\text{ss}}E_{\text{ss}}^{2}+b_{\text{nn}}E_{\text{nn}}^{2}\\ +b_{\text{fs}}\left(E_{\text{fs}}^{2}+E_{\text{sf}}^{2}\right)+b_{\text{fn}}\left(E_{\text{fn}}^{2}+E_{\text{nf}}^{2}\right)\\ +b_{\text{ns}}(E_{\text{ns}}^{2}+E_{\text{sn}}^{2}), \end{array}$$

where *E*_ij_ (*i, j* = *f, s, n*) are components of the Green-Lagrange strain tensor expressed in a reference frame locally aligned along the fiber direction (*f*), the orthogonal direction spanning the myocardial sheet (*s*), and the cross-fiber direction (*n*). Values for the *C* and *b*_ij_ (*i, j* = *f, s, n*) coefficients are reported in Table 1.

**Table 1:** Sets of material properties considered in the study.

	**Reference**	***C*, *a***	***b***	***a*_ff_**	***b*_*ff*_**	***a*_*ss*_**	***b*_*ss*_**	***a*_*fs*_**	***b*_*fs*_**	***b*_*n*[*n, f, s*]_**
	**(species)**	**(kPa)**		**(kPa)**		**(kPa)**		**(kPa)**		
*W*_*U*_	Usyk et al. (2000) (canine)	0.88			8.00		6.00		12.00	3.00
$W_{HO}^{W}$	Wang et al. (2013)(swine)	0.24	10.81	20.04	14.2	3.72	5.16	0.41	11.30	
$W_{HO}^{G}$	Gültekin et al. (2016) (human)	0.4	6.55	3.05	29.05	1.25	36.65	0.15	6.28	

*W_U_ is expressed in terms of components of the Green-Lagrange strain tensor **E**, while $W_{HO}^{\left\{W,G\right\}}$ depends on invariants of the right Cauchy-Green tensor **C***.

The remaining 2 constitutive behaviors here considered followed the constitutive law based on the invariants of the right Cauchy-Green strain tensor **C** proposed by Holzapfel and Ogden (2009),

(4) $$\begin{array}{l} W_{HO}=\frac{a}{2b}\left\{\exp\left[b(I_{1}-3)\right]\right\}+\sum_{i\;=\text{ ff},\text{ss}}\frac{a_{i}}{2b_{i}}\left\{\exp\left[b_{i}{\left(I_{4i}-1\right)}^{2}\right]-1\right\}\\ \;+\frac{a_{\text{fs}}}{2b_{\text{fs}}}\left\{\exp\left[b_{\text{fs}}I_{\text{8fs}}^{2}\right]-1\right\}, \end{array}$$

where *I*_1_=tr **C** is the first invariant of **C**, here applied as the argument of an exponential term; *I*_4*i*_ = **v**_*i*_ · (**C** · **v**_*i*_), *i* = ff, ss is the fourth invariant of **C**, which corresponds to the squared stretch of a line element oriented along the fiber (**v**_ff_) or sheet (**v**_ss_) directions; finally, *I*_8fs_ = **f**_0_ · (**C** · **s**_0_) is the eighth invariant of **C**, which captures the effects of strain coupling. Equation (4) has been shown to describe well experiments on pig ventricles (Dokos et al., 2002), and more recently the biaxial and triaxial tests conducted on human myocardial tissue by Sommer et al. (2015). Among best-fit values reported in literature, we selected materials parameters for (4) from Wang et al. (2013) ($W_{HO}^{W}$, fitted to experiments on swine models) and Gültekin et al. (2016) ($W_{HO}^{G}$, fitted to experiments on human tissue). The coefficients for all considered material properties are reported in Table 1.

### 2.3. FEM models of LV passive biomechanics

High-resolution FEM simulations of LV biomechanics are at the core of the parameter exploration and inverse estimation strategies presented in this work. To cope with the complexities of the mechanical behavior of the myocardium, we employed a recently validated numerical solver suitable for dealing with incompressible hyperelastic material laws such as those in (3) and (4) (Gurev et al., 2015), and extended to use stabilized P1/P1 finite elements. The capabilities are necessary for infarct simulations, where capturing sufficient detail at the border zone region around the lesion is pivotal (see section 2.6). The solution algorithm also allows multi-scale effects, and we used the TriSeg ODE-based model with parameters for human to drive myofilament active contraction (Lumens et al., 2009; Gurev et al., 2015). Coupling between cellular and tissue mechanics occurred at the Gauss point level.

To handle the relatively large number of simulations needed to train statistical models, we developed an automatic workflow to construct high-resolution computational domains from any given sets of geometric parameters ξ describing LV anatomy. In this pipeline, analytical models built according to (1) were first converted to 3-dimensional triangulated surfaces, and then to solid meshes of several hundred thousands of tetrahedral elements. Nodes at the base of the ventricle were prevented to move axially, while epicardial nodes in the vicinity of the base (i.e., closer than 3 mm) were fully locked to prevent rigid motions. Boundary traction effects from the pericardial membrane and the right ventricle were neglected, and intraventricular pressure was uniformly applied at the endocardial surface in quasi-static steps. The vector **v**_ff_ of alignment of myocardial fibers varies heterogeneously along the radial direction of the myocardium (McCulloch, 1999; Humphrey, 2002). Without specific measurements for the patients in the database, we relied on a rule-based approach to assign fiber directions linearly varying their angle with respect to the circumferential direction from 90° at the endocardial surface (i.e., longitudinally aligned) to -60° at the epicardium.

The mechanical equilibrium equations were solved in parallel on the Cognitive Computing Cluster (CCC), a hybrid high performance shared resource developed at IBM Research deploying both Intel and Power8 nodes. Active infarct simulations required ~10 times more resources than passive models, and were run on the Uran Supercomputer hosted by UB RAS and Ural Federal University. Outputs of the simulations were nodal displacement vector fields, and components of stress and strain tensors defined at the element Gauss points. To relate predictions also to strain dependent length activation of the sarcomere, we also evaluated stretch in the fiber direction, defined as

(5) $$\lambda_{\text{ff}}=\sqrt{v_{\text{ff}}\cdot C\cdot v_{\text{ff}}},$$

where ***v***_ff_ is the vector aligned along the myofiber direction (as described above), and **C** is the right Cauchy-Green strain tensor. As a representative scalar of each loading state, we also averaged λ_ff_ at midwall, which we defined as a tissue slab located between 40 and 60% of the LV wall thickness and between 45 and 55% of the apex-base distance.

### 2.4. Parameterization of FEM results

A key aspect of the inverse unloading method presented in this work is the re-parameterization of FEM simulation results in terms of the same geometric parameters employed to process the Sunnybrook database. A 2-step optimization procedure was implemented to fit idealized models of LV anatomy to the deformed configurations predicted by the FEM analyses upon varying loading conditions. First, optimal values for *R*_*b*_, *Z*, *e*, and Ψ_0_ were found to minimize average nodal distance between the profile of an idealized epicardium and the corresponding boundary obtained from a FE mesh warped according to the simulations results. Second, a similarly defined nodal distance measure was used to quantify discrepancies between endocardial profiles in order to adjust the remaining *L* and *H* parameters. The 2 steps were re-iterated until reaching convergence. An alternative monolithic approach where the 6 parameters were optimized at the same time was also evaluated, but proved to be less computationally efficient.

### 2.5. Statistical learning of LV unloading

**Figure 2:**
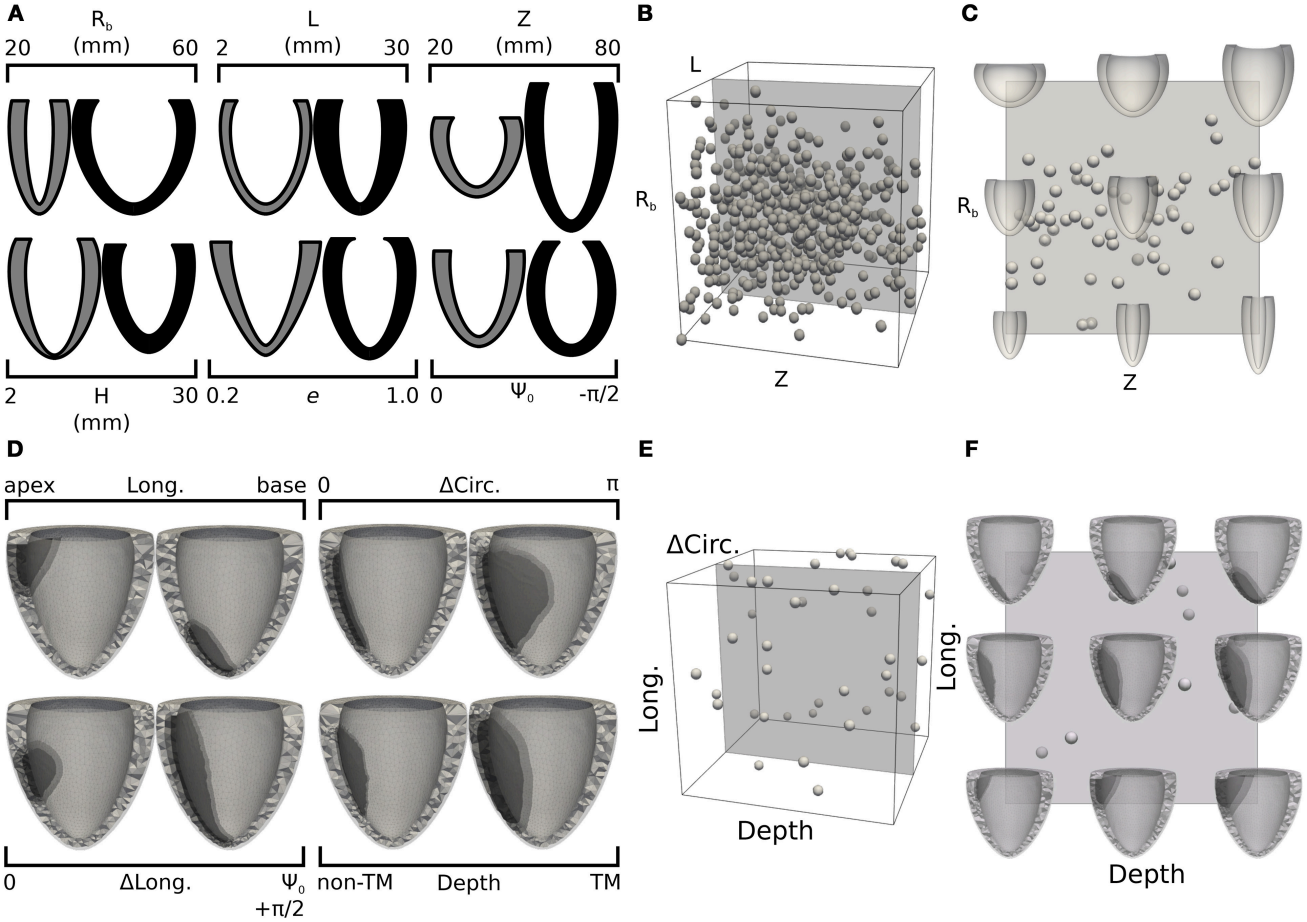
Design of training sets for the 2 statistical models: LV unloading **(A–C)** and infarct shape effects **(D–F)**. **(A)** Pairs of LV cross-sections representing extreme geometries limiting parameter space dimensions. Gray (black) cross-sections correspond to extreme negative (positive) variations of one of the geometric parameters, with the remaining 5 parameters kept at mid-range values. **(B)** Projection of the 6-D parametric space onto a 3-D cube obtained by neglecting the last 3 dimensions (H, *e*, and Ψ_0_). Spherical glyphs indicate locations of 600 sampling points chosen via latin hypercube sampling from a normal distribution centered on the average LV geometry and with a doubled standard deviation compared to that of the complete Sunnybrook database. **(C)** Cross-section of the parameter space for LV unloading showing combined variations of R_*b*_ and Z parameters. **(D)** Similar to **(A)**, but showing pairs of FE meshes including infarct regions with extreme shapes. The lightest tone of gray indicates the healthy region, the darkest tone indicates the infarct, and the intermediate one marks the refined border zone. **(E)** 3-D projection of the 4-D parameter space defining infarct shape obtained neglecting the ΔLong. dimension. Similarly to **(B)**, spheres indicate locations of 40 sampling points chosen uniformly in the allowed range parameters. **(F)** Mid-range slice of the 3-D projection showing representative FE meshes accounting for combined variations of longitudinal location and transmural depth of the infarct.

Bulk processing the Sunnybrook cine-MRI image datasets provided information on expected anatomical variability among patients. As part of our inverse unloading estimation strategy, we leveraged database statistics to define a 6-D parameteric space that enclosed all likely LV unloaded configurations. More specifically, we reasoned that the parametric study should conservatively admit and explore large variations in ventricle geometries, since the unloaded state might differ significantly from any of the imaged configurations. Limits of the parametric space were therefore defined to encompass variations of more than 3 standard deviations from the average beginning of diastole (BoD) state, which we chose as most reasonable guess lacking the measurements needed for better estimates (e.g., Xi et al., 2013). More details on the subdivision of the cardiac cycle into its phases are reported in the Supplemental Material. Figure 2A shows pairs of limit parameter values and corresponding LV cross-sections representing maximum allowed variations of each of the 6 geometric features. In drawing the profiles, only one of the 6 parameters was changed while keeping the remaining 5 at corresponding mid-range values. Unloaded configurations admitted to our study were, therefore, intermediate states of the low- and high-parameter geometries shown in Figure 2A in gray and black tones, respectively. The statistical distribution of BoD states was also used to design an efficient probing scheme for the parametric space defined above. As is common now (Marsden, 2014), we used latin hypercube sampling to select 600 points (i.e., 100 times the number of parameters) from a normal distribution centered on the average BoD state and with doubled standard deviation compared to that of the Sunnybrook database. Samples falling beyond the limits defined in Figure 2A were projected onto the closest admissible point. A cloud of chosen probing locations is shown within a unitary 3-D projection of the parameter space in Figure 2B. For this representation, the *H*, *e*, and Ψ_0_ dimensions were neglected. Figure 2C further shows a mid-slice of the parametric cube exploring geometries corresponding to coupled variations of the *R*_*b*_ and *Z* parameters.

For each of the 600 sampled ventricle geometries we ran passive inflation simulations for inner LV pressures ranging between 0 and 5 kPa. Results were processed as described in section 2.4 to find optimal geometric parameters for 100 intermediate loading configurations (i.e., differing by 0.05 kPa). These best-fit parameters constituted the training set for GP regression models mapping loaded configurations to their corresponding unloaded state. Overall, we optimized 100 statistical models (one for each considered inner pressure), and fitted 2 additional GP regressions for unloading the inflated configurations for which the midwall fiber stretch reached the values of 10 and 15%.

### 2.6. Statistical learning of infarct shape and size on LV performance

With our solver capable of handling high-resolution tetrahedral meshes, we explored the effects of different infarct shapes and locations on simulated LV cardiac cycles. The lesions were parameterized according to 4 features: longitudinal position (Long. ∈ [0, 1]), indicating whether an infarct was closer to the base (Long.=0) or apex (Long.=1); circumferential extension (ΔCirc. ∈ [0, π]), indicating the portion of circumference (measured in radians) occupied by the infarct; longitudinal extension (ΔLong. ∈ [0, 1]) indicating the fraction of longitudinal cross section harboring a lesion; and wall depth (Depth ∈ [0, 1]), indicating the transmural extension of the infarct, with the maximum value of 1 indicating a fully transmural lesion. Figure 2D shows computational domains reconstructed from limit cases of the infarct parameterization. Similar to that presented in section 2.5, latin hypercube sampling was used to efficiently probe the parameter space. Our sample size was of 40 points, (i.e., 10 times the number of parameters), and we assumed a uniform distribution of parameters across the admissible range. Also, to restrict our attention to the effects of infarct without the added complications introduced by changing LV geometry, all lesions were inserted into the same baseline LV from patient I-02. Infarct effects were simulated by deactivating active contraction in the lesion regions, while maintaining the same passive material properties. Similar to Figures 2B,C,E,F show projections of selected samples onto the considered parameter space of infarct lesions. More details on the general procedure followed to mesh infarcted regions of arbitrary shapes are available in the Supplemental Material.

## 3. Results

**Figure 3:**
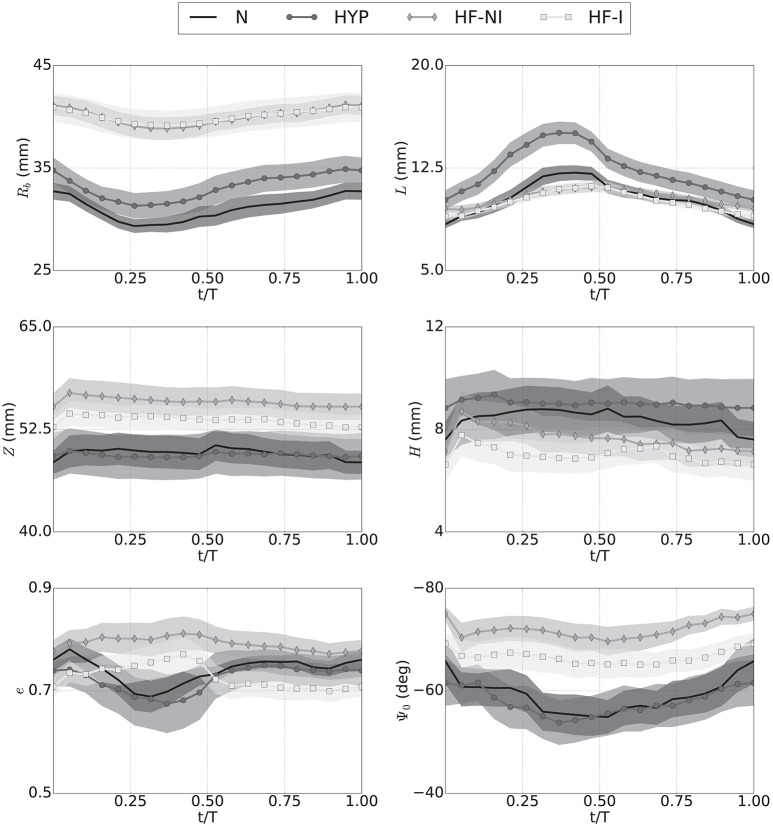
Kinematics of LV motion during a normalized cardiac cycle described by changes over time to the 6 parameters of an idealized model of LV geometry. Traces show averages (marked solid line) over the 4 patient groups (N, normal; HYP, hypertrophic; HF-NI, heart failure without infarct; HF-I, heart failure with infarct) plus or minus standard deviations (shown as semi-transparent overlays). Each subplots shows results for one of the geometric parameters. Results were obtained by custom procedure to fit idealized model to segmentation results. See text for more detail.

Once enhanced with rigid motion correction, the 6-parameter description of LV geometry was able to capture anatomical and kinematic features from the Sunnybrook MRI scans. Median values of the similarity functional *J*(*I*_**ξ**_, *I*_MR_) averaged for each category of patients were 0.29 for N, 0.30 for HYP, 0.23 for HF-NI and 0.19 for HF-I, respectively. Figure 3 shows average group traces of best-fit geometric parameters (see Equation 1) over the course of a normalized cardiac cycle. Certain trends agreed well with known morphologic features of cardiac disease. Patients affected by heart failure (i.e., from the HF-I and HF-NI categories) presented on average the most dilated ventricles, as indicated by the largest *R*_*b*_ values, and the smallest cyclical variations in both *e* and Ψ_0_, probably due to myocardial dysfunction. Hypertrophic patients, on the other hand, maintained highest *L* values throughout the cycle (*L* = 12 mm on average) and showed a large systolic thickening (*L* = 15 mm at peak systole for HYP patients). Only N subjects contracted more visibly, with an average 54% increase in *L* from diastole to systole. N and HYP subjects overall exhibited the largest changes in truncation angles. Other parameterization findings were less intuitive. For all LVs, contraction in the longitudinal direction was captured mainly by varying Ψ_0_ rather than *Z*, which instead remained close to constant throughout the cycle. Also, the dynamic pattern of *e* observed in HF patients was peculiar. For example, 10 out of 12 HF-I subjects exhibited increased *e* at systole compared to diastole, while the opposite was typically observed in the N and HYP categories of patients. Combined behavior of *e* and Ψ_0_ differed also among the 2 classes of HF patients: in presence of an infarct, both *e* and Ψ_0_ were smaller in magnitude, indicating that HF-NI ventricles tended to be more spherical than the HF-I ones. Table 2 reports best fit sets of geometric parameters for all 45 patients at both beginning and end of diastole (BoD and EoD, respectively).

**Table 2:** Best-fit geometric parameters for all patients in correspondence of beginning and end of diastole configuration (BoD, and EoD, respectively).

	***R***_*****b*****_ **(mm)**		***L*** **(mm)**		***Z*** **(mm)**		***H*** **(mm)**		***e***		Ψ_**0**_ (^****°****^)	
	**EoD**	**BoD**	**EoD**	**BoD**	**EoD**	**BoD**	**EoD**	**BoD**	**EoD**	**BoD**	**EoD**	**BoD**
N-02	33	29	8.1	11	49	49	8.4	7.9	0.71	0.64	−74	−77
N-03	33	30	7.5	13	46	45	5.4	5.6	0.75	0.78	−79	−56
N-05	30	26	8.1	12	43	44	6.9	6.3	0.7	0.71	−61	−48
N-06	32	29	7.3	11	44	44	5.6	5.2	0.87	0.8	−69	−58
N-07	34	30	9.3	12	54	55	11	12	0.7	0.6	−60	−44
N-09	38	32	8.2	12	47	51	9.1	13	0.72	0.54	−78	−66
N-10	33	30	10	17	56	63	5.4	11	0.73	0.69	−80	−56
N-11	34	31	8.7	14	48	49	6.6	7.2	0.8	0.68	−50	−52
N-40	29	26	8.1	11	49	50	9.7	10	0.87	0.81	−40	−34
HYP-01	32	27	7.1	12	39	40	5.3	6.4	0.64	0.46	−79	−70
HYP-03	34	33	9.5	18	42	47	6.1	8.0	0.6	0.55	−72	−43
HYP-06	34	29	9.3	12	38	37	6.4	5.6	0.75	0.68	−76	−76
HYP-07	39	35	12	18	53	57	7.9	7.3	0.96	0.89	−50	−47
HYP-08	44	41	14	20	65	64	11	9.6	0.82	0.93	−61	−42
HYP-09	35	32	7.9	13	54	45	7.5	9.6	0.82	0.7	−76	−68
HYP-10	40	36	9.0	13	46	45	5.3	5.1	0.69	0.56	−61	−56
HYP-11	31	29	9.7	14	37	33	6.6	5.1	0.83	0.98	−79	−79
HYP-12	28	24	7.1	12	49	52	8.3	11	0.7	0.43	−66	−49
HYP-37	36	32	11	17	51	54	13	12	0.67	0.55	−33	−28
HYP-38	34	30	13	17	68	64	19	16	0.66	0.52	−45	−53
HYP-40	32	30	13	16	50	50	10	10	0.8	0.85	−41	−39
HF-NI-03	46	44	11	13	52	54	6.3	6.5	0.88	0.96	−78	−60
HF-NI-04	42	40	8.4	13	49	49	5.9	5.2	0.71	0.69	−73	−58
HF-NI-07	39	37	8.8	11	64	62	12	10	0.69	0.75	−68	−73
HF-NI-11	44	42	9.7	10	59	56	5.7	5.2	0.69	0.67	−79	−77
HF-NI-12	47	44	8.7	11	62	61	7	6.7	0.79	0.87	−78	−74
HF-NI-13	41	40	9.7	12	62	64	8.4	9.5	0.9	0.88	−80	−79
HF-NI-14	40	37	11	12	53	55	7.6	10	0.79	0.82	−68	−62
HF-NI-15	36	32	9.3	9.7	56	57	12	12	0.81	0.92	−64	−58
HF-NI-31	40	35	9.4	11	49	49	5.8	5.2	0.84	0.98	−78	−79
HF-NI-33	37	34	9.2	12	57	55	6.7	6.2	0.7	0.64	−80	−77
HF-NI-34	40	39	9.4	13	58	63	5.1	10	0.71	0.72	−71	−49
HF-NI-36	43	41	8.5	9.3	45	44	5.2	5.4	0.79	0.77	−77	−79
HF-I-01	38	36	8.3	9.4	54	54	5.1	5.1	0.84	0.95	−64	−67
HF-I-02	44	40	9.5	10	52	53	5.7	5.9	0.65	0.65	−75	−75
HF-I-04	41	40	8.8	11	50	51	5.7	5.3	0.66	0.66	−63	−55
HF-I-05	41	38	9.4	11	48	54	8.9	11	0.68	0.83	−67	−48
HF-I-06	39	38	8.5	11	54	57	5.3	5.3	0.7	0.84	−77	−75
HF-I-07	38	37	10	14	42	43	6.8	8.1	0.57	0.46	−71	−70
HF-I-08	42	41	9.4	11	54	54	5.4	5.2	0.77	0.77	−59	−57
HF-I-09	51	50	10	11	65	64	5.4	5.2	0.74	0.73	−72	−69
HF-I-10	49	47	9.2	10	53	58	5.1	9.0	0.74	0.83	−73	−79
HF-I-11	40	39	7.2	9.4	55	54	5.9	5.0	0.68	0.81	−59	−61
HF-I-12	36	34	8.4	15	54	56	7.6	7.2	0.69	0.74	−67	−50
HF-I-40	33	31	8.2	12	54	51	13	10	0.76	0.88	−80	−78
Avg. N	33 ± 3	29 ± 2	8.4 ± 0.9	13 ± 2	48 ± 4	50 ± 6	7.6 ± 2.1	8.7 ± 2.9	0.76 ± 0.07	0.69 ± 0.09	−66 ± 14	−55 ± 12
Avg. HYP	35 ± 4	32 ± 5	10 ± 2	15 ± 3	49 ± 10	49 ± 10	8.9 ± 4.0	8.8 ± 3.2	0.74 ± 0.10	0.67 ± 0.19	−62 ± 16	−54 ± 16
Avg. HF-NI	41 ± 3	39 ± 4	9.4 ± 0.9	11 ± 1	56 ± 6	56 ± 6.2	7.3 ± 2.4	7.7 ± 2.5	0.77 ± 0.08	0.81 ± 0.12	−74 ± 6	−69 ± 11.0
Avg. HF-I	41 ± 5	39 ± 5	8.9 ± 0.8	11 ± 2	53 ± 5	54 ± 5	6.7 ± 2.3	6.9 ± 2.2	0.71 ± 0.07	0.76 ± 0.13	−69 ± 7	−65 ± 11

**Figure 4:**
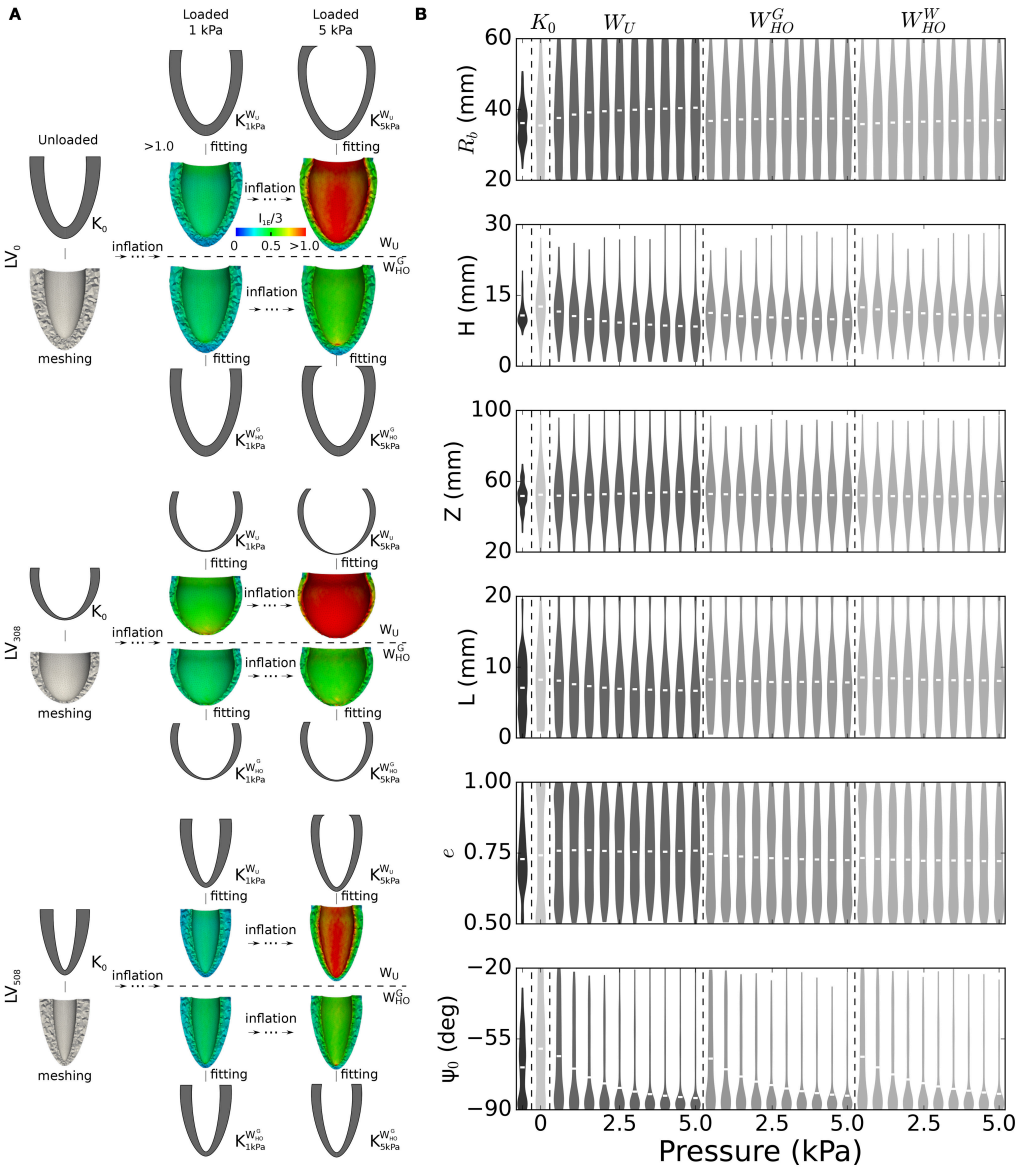
Generation of training dataset for the unloading problem. **(A)** Passive inflation and subsequent parameterization results for selected sample of 3 out of 600 left ventricle geometries considered to build the training datasets for the unloading problem. Idealized geometries, chosen via latin hypercube sampling to probe the parameter space, were discretized and subjected to passive inflation using 3 different sets of material properties. Shown in the panel are results for 2 sets of material properties (*W*_*U*_ and $W_{HO}^{G}$) and 2 loading pressures (1 and 5 kPa). Shown also are color-coded distributions of strain expressed as the first invariant of the Green-Lagrange tensor. **(B)** Violin plots depicting changes in geometric parameter distributions upon inflation for all the 533 LV geometries included in the training datasets, and for the 3 sets of material properties. Black tone plots indicate distributions of geometric parameters at the BoD configuration. Lightest gray tone plots correspond to distributions synthesized via latin hypercube sampling from a normal distribution constructed based on the BoD configuration, but allowing 2-times larger variations. White segments close to the center of the distribution indicate mean values. See text for more details.

The distribution of LV shapes at BoD (see Table 2) was pivotal to design our admissible parameter space, both for establishing range limits and for choosing the frequency of allowed variations. Figure 4A shows 3 representative unloaded configurations out of the 600 selected to probe the space. Each geometry was first discretized into a computational domain (see meshes below the idealized profiles) and then inflated with inner pressures up to 5 kPa. Shown also are color coded distributions of the first invariant of the Green-Lagrange strain tensor (*I*_1*E*_). Strain fields were visibly larger in the LVs endowed with *W*_*U*_ material properties (i.e., those on the first row of each subgroup) than in those endowed with $W_{HO}^{G}$ (i.e., those on the second row of each subgroups). While the parameteric study extensively explored combined effects of LV geometric features on deformation, the subsequent processing step converted results back to the 6-parameter description (see profiles in gray above and below strain results). Out of the chosen 600 probing profiles, 67 exhibited incompatible features that prevented completion of the FEM simulations (e.g., a disproportionately large *L* and negative Ψ_0_ in a ventricle with minimum *R*_*b*_), and were therefore excluded from the analysis. Figure 4B shows violin plots of geometric parameter distributions for ventricles at the BoD configuration from the database, at the assumed unloaded configuration, and at 10 deformed configurations for pressures ranging from 0.5 to 5.0 kPa. The BoD distributions (see plots in black tone, leftmost sector) clearly reflected the categories of the database. For example, the violin plot of the *R*_*b*_ parameter (first row) indicated a bimodal distribution, as expected given the sharp differences in ventricle radius between HF patients and the others. By design, the sampled unloaded configurations followed a normal distribution allowing large variations (see plots in lightest gray tone, second sector from the left). Some hard limits on admissible parameter values were enforced to reduce the number of incompatible geometries selected (see section 2.5). The effects of these hard limits were noticeable particularly within the *L*, *e*, and Ψ_0_ distributions (see last 3 rows), the tails of which were thickened by assimilating parameters beyond allowed range. Finally, the distributions of loaded configurations (see plots in intermediate gray tones, three rightmost sectors) showed the evolution of geometric parameters upon pressurization, which followed the expected behavior for incompressible hyperelastic materials. For example, the *R*_*b*_ parameter increased relatively fast at low pressures, but then dilation progressively stopped accounting for the exponential increase in stiffness. The thickness parameters *L* and *H* decreased upon pressurization (also ensuring incompressibility), while the Ψ_0_ parameter distributions were the most sensitive to pressure. Finally, the material properties could be ranked in order of increasing stiffness as *W*_*U*_, $W_{HO}^{G}$, and $W_{HO}^{W}$, as shown by changes in mean values from the distributions (see white lines inside the violin plots).

**Figure 5:**
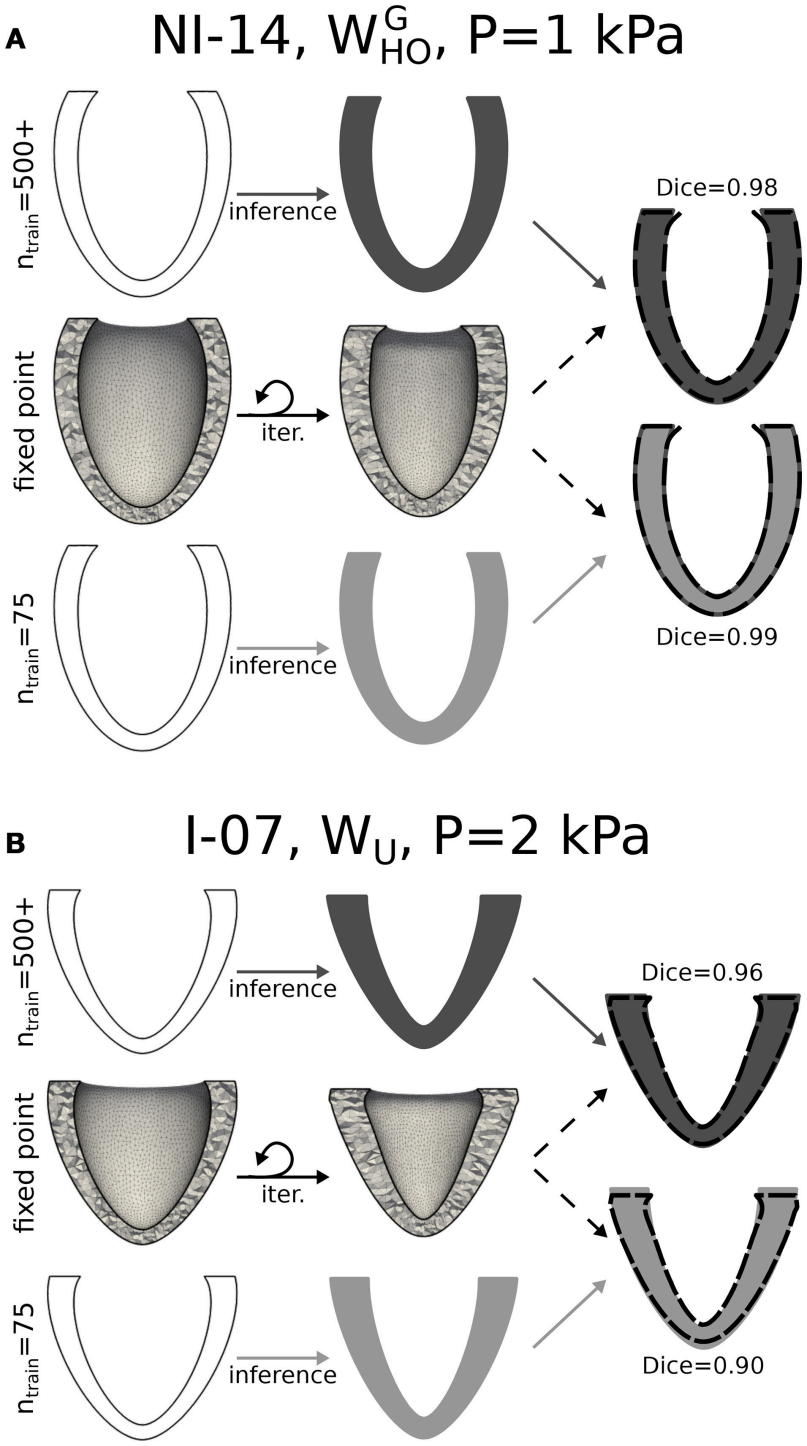
Unloading via kriging and comparison to the fixed point iteration method. **(A)** Unloading procedure is shown applied to a representative case (NI-14, unloading pressure P = 1 kPa, and $W_{HO}^{G}$ material properties) for which a statistical model trained on 75 arbitrary ventricles matched best unloading results via fixed point iteration method. While the fixed point iteration method required meshing of the ventricles in the loaded configuration and iterative updates (middle row), the statistical method allowed to infer the unloaded geometry directly from the 6 parameters describing the end-diastolic (loaded) configuration (bottom row). Top row is similar to bottom row, but shows result obtained after training a statistical model on results from the full parametric study of 500+ LVs. The rightmost column shows overlapped cross-sections of unloaded LVs obtained via the fixed point iteration method (dashed boundary) and 2 statistical models (solid gray tones). **(B)** Similar to **(A)**, but applied to another representative case (I-07, unloading pressure P = 2 kPa, and *W*_*U*_ material properties) for which the statistical learning method (with *n*_train_ = 75) yielded the worst overlap to fixed point iteration results (Dice score of 0.90). In this case, increasing the training set size led to improved results (Dice score of 0.96).

The computational cost of optimizing a GP regression to a few hundred training points (~1 CPU min) is negligible compared to that of running even only a single passive high resolution simulation. To optimize the use of computational resources, we sought, therefore, the minimum training set size that ensured satisfactory accuracy in estimating the unloaded configurations for all patients in the dataset. Figures 5A,B show cases where predictions by GP regression compared best (see Figure 5A) and least well (see Figure 5B) to the configurations predicted via fixed point iteration for a relatively small training size (*n*_train_=75). As starting (loaded) configurations, we chose geometries from the database at EoD (see first column in both panels), and from these we inferred corresponding unloaded configurations assuming inner LV pressures of either 1 or 2 kPa. Comparison between results from the 2 methods were evaluated in terms of Dice score between unloaded profiles (see Supplemental Material for details on Dice score computations). According to our analysis, *n*_train_=75 was the minimum training set size ensuring Dice scores larger or equal than 0.90 for all cases considered (i.e., including all the LV geometries, both EoD inner pressures, and the 3 sets of material properties). From the last column of Figure 5B one can appreciate how even a Dice score of 0.90 corresponds to a visibly good match between the GP regression prediction (see LV in gray tone) and corresponding geometry obtained via fixed point iteration (see overlapped dashed line). Small mismatches could be observed even in cases with high Dice score in regions close to the base of the LV (e.g., see last column of Figure 5A). These artifacts could be attributed to the zero-displacement boundary condition applied to epicardial elements within 3 mm from the base in the fixed point optimizations. Note that the fixed point iteration method required discretization of the EoD domain and repeated mesh deformation steps (see middle row in both panels). In contrast, after GP regression training the unloaded configurations could be inferred almost in real time, and as another advantage, the GP regression method eliminates potential issues introduced by iteratively warping the mesh (e.g., element degeneration) in the fixed point iteration method. The top row in both panels shows unloaded profiles inferred from GP regressions trained on the full set of simulation results. In the best match case shown in panel A results were essentially the same for *n*_train_=75, while the Dice score increased by 0.06 when we expanded the training dataset from *n*_train_=75 to 533 in the worst match case (see Figure 5B).

**Figure 6:**
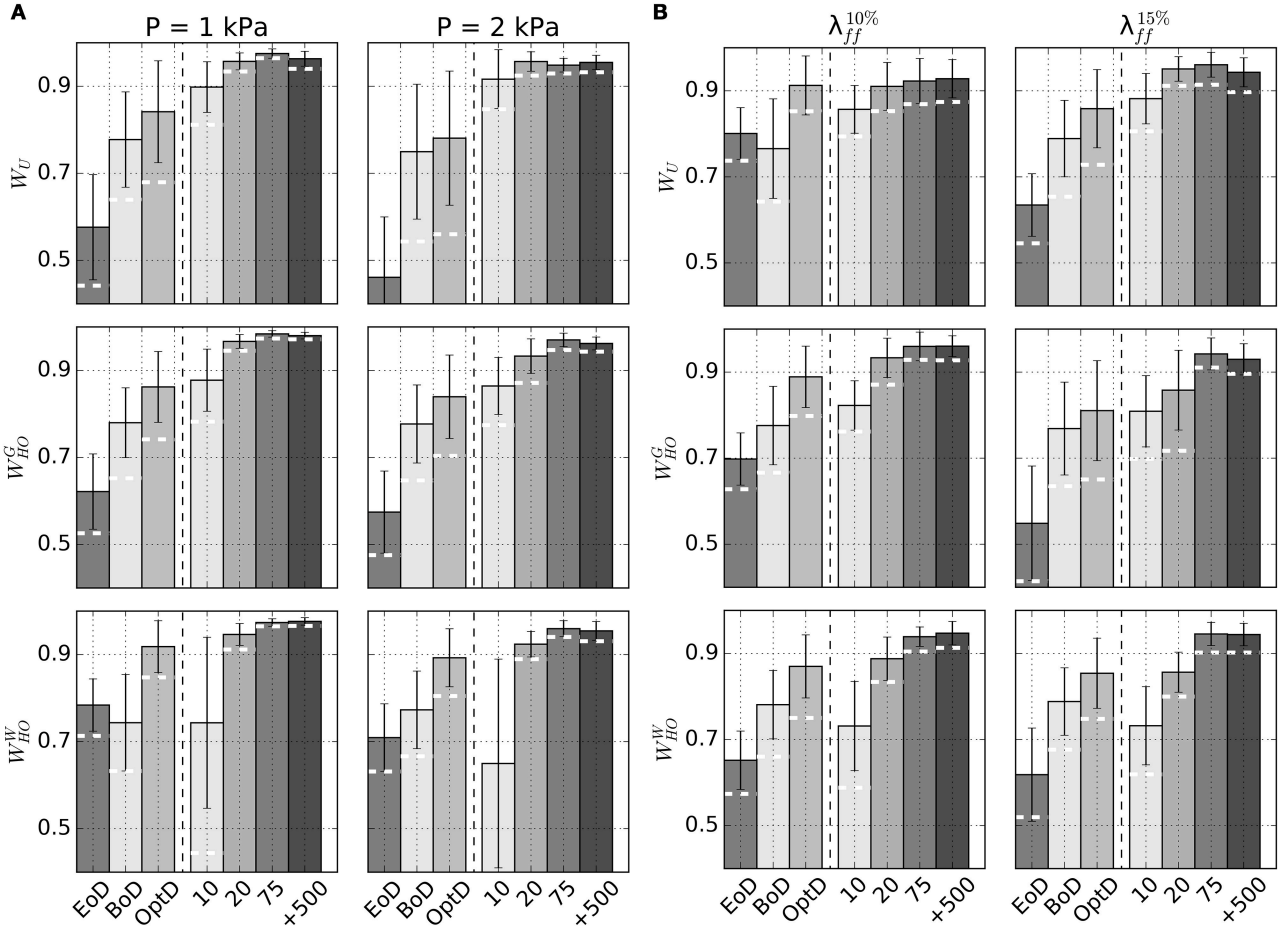
Accuracy of unloading via GP regression compared to unloading via fixed point iteration method. **(A)** Barplots of average Dice scores comparing beginning of diastole (BoD), end of diastole (EoD), the best-matching diastolic configuration (OptD), and unloaded configurations obtained via kriging with different *n*_train_ to unloaded configurations predicted via the fixed point iteration method. Subplots show results for combinations of considered material properties (*W*_*U*_ , $W_{HO}^{G}$, or $W_{HO}^{W}$) and unloading pressures (P = 1 kPa or 2 kPa). **(B)** Similar to **(A)**, but unloaded configurations are estimated prescribing average midwall strain at end diastole ($\lambda_{\text{f}{\text{f}}^{10\text{\%}}}$ on the left, $\lambda_{\text{f}{\text{f}}^{15\text{\%}}}$ in the right column). In both panels the dashed white lines drawn on kriging-related bars indicate lower 10th-percentile Dice score for each subcategory.

Figure 6 plots average Dice scores comparing GP regressions to fixed point iteration. The 3 rows in Figure 6A show results for different sets of material properties at an unloading pressure of 1 kPa (first column) or 2 kPa (second column). As expected, increasing training sizes generally yielded better Dice scores, although little improvement was observed beyond *n*_train_=75. Also reported are average Dice scores quantifying the overlap between fixed point iteration and the BoD or EoD configurations, as well as the average overlap with the OptD configuration, which was chosen as the imaged diastolic configuration that matched best the unloaded geometry. White dashed lines overlapped to the bars indicate the lower 10th-percentile Dice score observed for predictions from GP regressions.

Additional GP regression models were trained to handle situations where intraventricular pressure is unknown, but can be estimated by indirect measurements such as the fiber strain at midwall (see section 2.3). Table 3 reports unloaded geometries for all patients in the Sunnybrook database under the assumptions of *W*_*U*_ material properties and end-diastolic fiber strains of either 1.10 ($\lambda_{\text{ff}}^{10\%}$) or 1.15 ($\lambda_{\text{ff}}^{15\%}$). Outputs of the procedure included end-diastolic LV pressure values corresponding to the target fiber strains in the loaded configurations. Figure 6B reports accuracy of GP regression predictions measured in terms of Dice score with predictions via fixed-point iteration method.

**Table 3:** Unloaded geometries inferred via GP regression assuming EoD fiber stretches at midwall of either 1.10 ($\lambda_{\text{ff}}^{10\%}$) or 1.15 ($\lambda_{\text{ff}}^{10\%}$) and *W*_*U*_ set of material properties.

	***R***_*****b*****_ **(mm)**		***L*** **(mm)**		***Z*** **(mm)**		***H*** **(mm)**		***e***		Ψ_**0**_ (^**°**^)	
**Pat**.	**${\text{λ}}_{\text{f}\text{f}10}^{\%}$**	**${\text{λ}}_{\text{f}\text{f}}^{15\%}$**	**${\text{λ}}_{\text{f}\text{f}}^{10\text{\%}}$**	**${\text{λ}}_{\text{f}\text{f}}^{15\text{\%}}$**	**${\text{λ}}_{\text{f}\text{f}}^{10\text{\%}}$**	**${\text{λ}}_{\text{f}\text{f}}^{15\text{\%}}$**	**${\text{λ}}_{\text{f}\text{f}}^{10\text{\%}}$**	**${\text{λ}}_{\text{f}\text{f}}^{15\text{\%}}$**	**${\text{λ}}_{\text{f}\text{f}}^{10\text{\%}}$**	**${\text{λ}}_{\text{f}\text{f}}^{15\text{\%}}$**	**${\text{λ}}_{\text{f}}^{\text{f}10\text{\%}}$**	**${\text{λ}}_{\text{f}\text{f}}^{15\%}$**
N-02	30	30	9.3	10	50	51	9.1	9.8	0.65	0.65	−68	−60
N-03	30	29	8.4	9.4	47	47	6	6.5	0.70	0.71	−74	−66
N-05	28	28	9.3	10	45	46	7.6	8.1	0.65	0.65	−53	−45
N-06	29	29	8.4	9.3	45	45	6	6.6	0.86	0.89	−60	−53
N-07	32	32	11	12	56	58	12	13	0.63	0.63	−53	−45
N-09	35	34	9.6	11	54	55	12	13	0.70	0.70	−53	−45
N-10	30	30	11	12	58	58	5.6	6.1	0.69	0.71	−75	−68
N-11	32	32	10	11	49	50	7.4	7.9	0.76	0.78	−43	−35
N-40	28	28	9.5	10	51	52	10	11	0.83	0.84	−33	−27
HYP-01	30	30	7.9	8.9	39	39	6.2	6.8	0.57	0.56	−73	−64
HYP-03	32	32	11	12	43	43	6.9	7.6	0.54	0.54	−64	−55
HYP-06	32	32	11	12	38	39	7.4	8.1	0.73	0.75	−64	−57
HYP-07	37	36	14	15	56	57	8.8	9.3	1.00	1.00	−37	−31
HYP-08	41	41	16	17	68	70	11	12	0.82	0.84	−51	−44
HYP-09	33	32	9	9.7	55	55	8	8.7	0.80	0.81	−70	−63
HYP-10	36	35	9.5	10	46	46	6.2	6.9	0.58	0.57	−75	−66
HYP-11	29	29	12	13	38	39	7.3	7.9	0.83	0.86	−67	−60
HYP-12	26	26	8.1	9.1	51	51	8.7	9.4	0.65	0.65	−60	−53
HYP-37	31	32	11	12	53	54	13	14	0.55	0.55	−48	−40
HYP-38	32	33	14	15	71	72	17	17	0.61	0.60	−38	−32
HYP-40	31	31	15	16	53	54	11	12	0.78	0.80	−31	−24
HF-NI-03	43	42	12	13	52	53	7.2	8	0.89	0.91	−60	−53
HF-NI-04	39	38	9.3	10	49	49	6.9	7.7	0.66	0.66	−73	−64
HF-NI-07	36	35	9.4	10	66	67	14	14	0.59	0.58	−61	−54
HF-NI-11	40	39	11	12	60	60	6.4	7	0.63	0.63	−76	−68
HF-NI-12	43	41	9.6	10	63	63	8.4	9.2	0.77	0.77	−73	−67
HF-NI-13	38	37	11	11	63	63	9	9.6	0.89	0.90	−74	−68
HF-NI-14	37	36	12	14	54	55	8.3	9.1	0.77	0.79	−58	−50
HF-NI-15	34	34	11	12	60	61	13	14	0.85	0.86	−48	−41
HF-NI-31	37	36	11	12	50	50	6.6	7.2	0.84	0.86	−69	−62
HF-NI-33	34	33	10	11	58	58	7.3	8	0.64	0.64	−76	−69
HF-NI-34	37	36	10	11	59	60	5.6	6.3	0.65	0.65	−67	−59
HF-NI-36	40	39	9.4	10	45	46	5.9	6.6	0.82	0.83	−65	−58
HF-I-01	35	34	9.4	10	55	56	5.5	6	0.82	0.84	−57	−50
HF-I-02	41	39	11	12	53	53	6.6	7.3	0.59	0.58	−70	−61
HF-I-04	38	38	9.8	11	50	51	6.6	7.3	0.59	0.58	−57	−49
HF-I-05	39	38	11	12	49	50	10	11	0.61	0.60	−59	−50
HF-I-06	36	35	9.4	10	55	55	5.9	6.5	0.64	0.64	−74	−66
HF-I-07	36	36	12	14	42	43	7.8	8.6	0.50	0.50	−62	−53
HF-I-08	39	38	10	12	55	55	5.6	6.3	0.74	0.75	−58	−50
HF-I-09	47	45	10	12	74	74	13	14	0.73	0.73	−68	−62
HF-I-10	46	44	10	11	52	53	5.9	6.7	0.70	0.70	−66	−58
HF-I-11	36	35	7.1	7.8	54	54	5.7	6.4	0.60	0.59	−67	−59
HF-I-12	33	33	9.4	10	55	56	8.3	9	0.63	0.63	−62	−54
HF-I-40	31	30	9.6	10	55	56	13	14	0.71	0.70	−73	−65
N-avg	30	30	9.6	11	51	51	8.4	9.1	0.72	0.73	−57	−49
(±σ)	(2)	(2)	(1.0)	(1)	(5)	(5)	(2.5)	(2.7)	(0.08)	(0.09)	(14)	(14)
HYP-avg	32	32	12	12	51	52	9.3	10	0.71	0.71	−56	−49
(±σ)	(4)	(4)	(3)	(3)	(11)	(11)	(3.2)	(3)	(0.14)	(0.15)	(15)	(14)
HF-NI-avg	38	37	10	11	57	57	8.2	8.9	0.80	0.76	−67	−59
(±σ)	(3)	(3)	(1)	(1)	(7)	(7)	(2.7)	(2.6)	(0.1)	(0.12)	(9)	(9)
HF-I-avg	38	37	9.9	11	54	55	7.8	8.6	0.66	0.65	−64	−56
(±σ)	(5)	(4)	(1.2)	(2)	(7)	(7)	(2.8)	(2.9)	(0.09)	(0.09)	(6)	(6)

See text for more details

**Figure 7:**
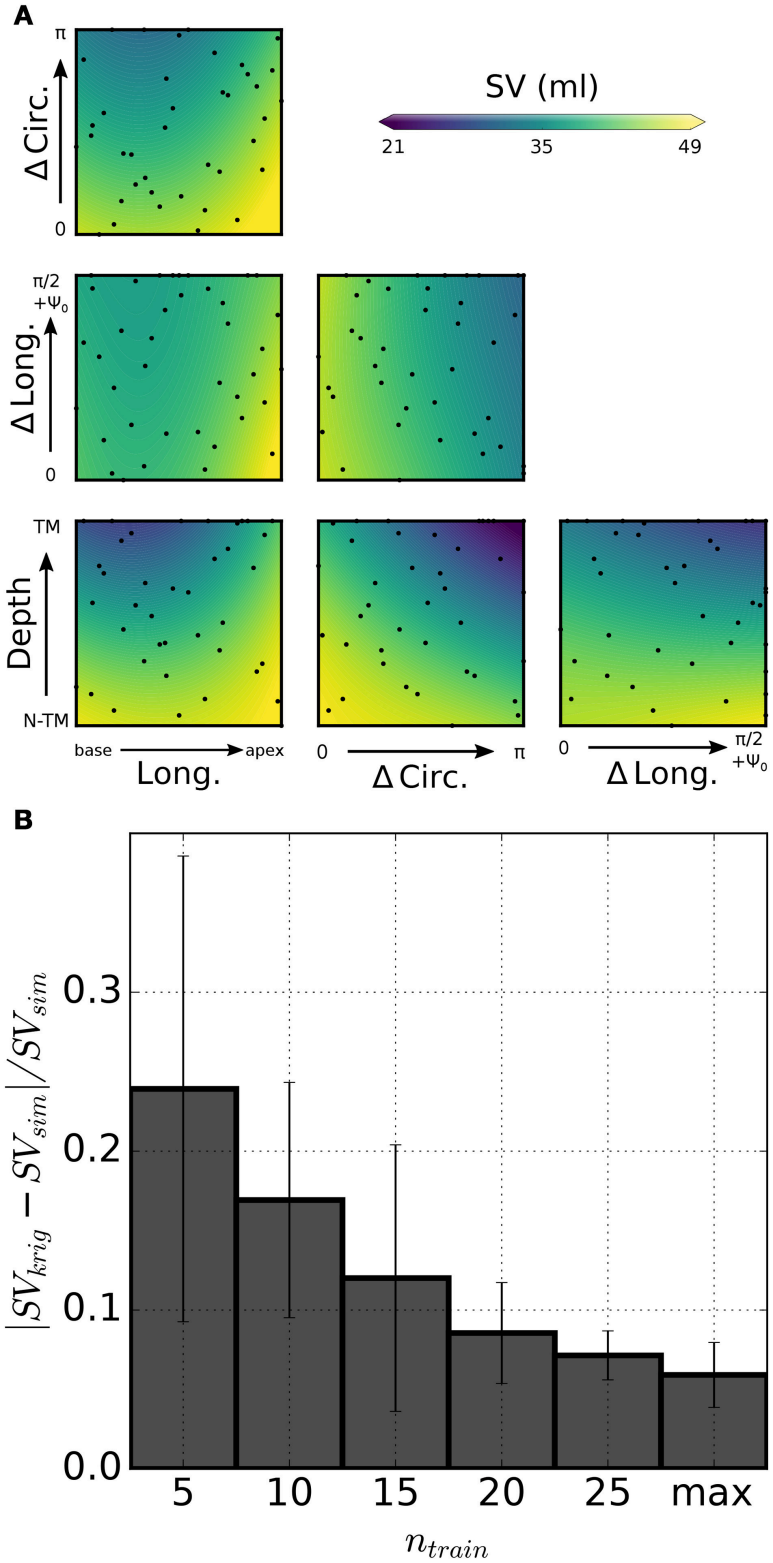
Statistical model of infarct shape and location effects on simulated SV. **(A)** Color-coded distribution of SV as predicted by kriging on 6 slices of the 4-D parametric hyperspace. Each plot shows combined effects of variations of 2 parameters on simulated SV, as shown by scale bar (values outside the range are truncated). Darker (lighter) color tones indicate stronger (weaker) impairment due to infarct. Dots represent projections of the probing points onto the slice plane. **(B)** 5-fold cross-validation to assess performance of the statistical model for varying training sizes *n*_train_. Relative error on simulated SV predictions approached 6% for the maximum training set size (*n*_train_ = 40).

Other than being used for inverse problems, GP regressions are ideal as tools for rapidly exploring multi-dimensional parameter spaces. As a proof of concept for the usage, we show preliminary results for a parametric study of the effect of infarct location and shape on cardiac performance as assessed by stroke volume (SV). Figure 7A shows color maps of simulated SV over 2-D slices of the 4-D parameteric space. Also shown, are projections onto each slice of the probing locations composing the full training set (see black dots). Each plot isolates the combined effects of 2 out of the 4 parameters used to define infarct shape and location. As expected, increases in lesion sizes yielded significant drops in SV. Maximum combined effect was reached by increasing both circumferential and transmural extension. Starting from a baseline failing LV with SV = 49 ml, GP regression predicted a drop down to SV = 21 mL at maximum depth and circumferential extension. Figure 7B shows 5-fold cross-validation for evaluating progressive convergence of GP regression for increasing training sizes. Average relative discrepancies between SV values from simulations and corresponding predictions from GP regression progressively decreased to 6% for a maximum training size of 40 simulations.

**Figure 8:**
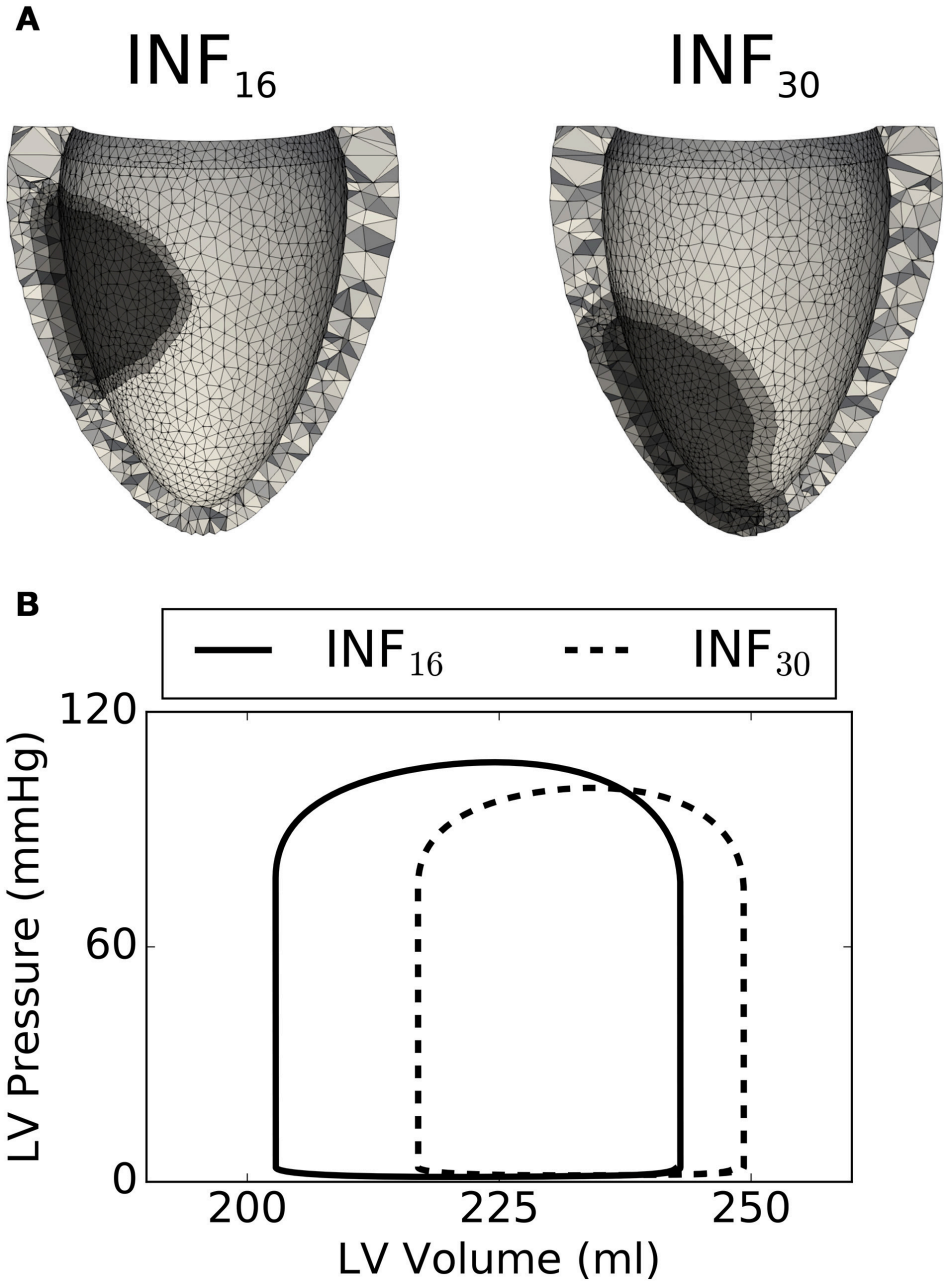
Comparison between 2 select simulations (out of the 40 considered). **(A)** On the left, the INF_16_ model has a smaller basal infarct (volume of 8.8 ml, Long. = 0.43, ΔCirc. = 1.22, ΔLong. = 0.40, Depth = 0.40). On the right, INF_30_ presents a larger transmural lesion (volume of 16 ml, Long. = 0.79, ΔCirc. = 1.57, ΔLong. = 0.46, Depth = 1.0). **(B)** Simulated PV loops showing smaller SV for the largest lesion INF_30_ (dashed line), as expected.

Figure 8A compares in detail 2 simulations from the training set characterized by different infarct morphologies. While INF_16_ (on the left) harbored a non-transmural basal infarct, the lesion in INF_30_ was larger, more apical, and fully transmural. The high level of mesh refinement within and surrounding the infarct (see regions in darkest and intermediate gray tones, respectively) required the capability of our solver of handling high resolution tetrahedral meshes. Figure 8B compares simulated PV loops for the 2 models described above. As expected, INF_30_ (see dashed line), which harbored a larger lesion, exhibited a stronger impairment in simulated cardiac performance. The PV loops show the weaker contraction generated by INF_30_ despite an increase in end-diastolic volume (i.e., SV = 40 ml and SV = 32 ml for INF_16_ and INF_30_, respectively).

## 4. Discussion

Numerous computational models of LV mechanics have been developed over the years to understand better LV function in normal and diseased hearts with the ultimate goal of assisting personalized diagnostics and treatment. Available models differ both in terms of enclosed biophysical detail and of anatomical representation. In the simplest form, left ventricular function can be captured by a time-varying elastance model, where a single time-varying ODE couples the evolution of intraventricular pressure and volume over the course of a cycle (Suga and Sagawa, 1972; Stergiopulos et al., 1996). At the other end of the complexity scale, models of LV mechanics incorporate phenomenological or biophysical descriptions of muscle contraction at the microscopic level, while at the same time capturing in detail the cardiac anatomy on high-resolution computational domains (e.g., Guccione et al., 1995; Kerckhoffs et al., 2007; Göktepe and Kuhl, 2010; Baillargeon et al., 2014; Sundnes et al., 2014; Gurev et al., 2015; Augustin et al., 2016). Although these highly refined 3D models provide valuable information, they entail high computational costs. To improve computational efficiency, models with intermediate levels of complexity have been based on simplifying assumptions on ventricular geometry and structure (Arts et al., 1979; Beyar and Sideman, 1984; Lumens et al., 2009). For prolate spheroid geometries and passive mechanics simulations, distributions of stress in other low order models can match well FEM results despite running faster than in real-time (Moulton and Secomb, 2013, 2014; Moulton et al., 2017).

Significant reductions in computational costs can be similarly achieved by training machine learning models on the results of opportunely sampled biophysical simulations. As a proof of concept, in this paper we applied GP regression, a popular supervised learning technique, to 2 problems of interest in cardiac mechanics modeling. First, 600 LV geometries described by a 6-parameter (*R*_*b*_, *L*, *Z*, *H*, Ψ_0_, *e*) prolate spheroid were extracted randomly from a conservatively defined parameter space. For each geometry, a forward simulation was run to trace ventricle geometries upon inflation at progressively larger intraventricular pressures. GP regression models then allowed to infer unloaded configurations given sets of 6 parameters defining the loaded geometries and either their corresponding intraventricular pressure or their fiber strain at midwall. For the second statistical model, we built a GP regression between parameters characterizing the location and shape of an infarct and corresponding stroke volumes predicted by high-resolution simulations accounting for the presence of the lesion.

### 4.1. Ventricular shape analysis

The Sunnybrook Cardiac MRI database was the primary source of imaging data for this study. Conventional analyses of the segmentations from such a database have employed methods to either extract features directly from images (e.g., Chumarnaya et al., 2016), or have used finite element models to analyze ventricular shapes and build statistical classifiers of patient disease (e.g., Piras et al., 2017). A geometric description with fewer parameters is better suited for parameterizing the geometry of ventricles in regressions trained on biophysical simulation results. Therefore, instead of finite element models, we adopted a 6-parameter description (Streeter and Hanna, 1973; Pravdin et al., 2014) to approximate ventricular geometry. In spite of its simplicity, this approach was able to capture some of the shape features and biomarkers that have been previously extracted using the conventional finite element models (e.g., Zhang et al., 2014). In particular, ventricular sphericity (*e*) separated ventricles with and without myocardial infarction in patients with heart failure (see HF-I and HF-NI traces in Figure 3). The 6-parameter model analysis also captured higher average wall thickness in hypertrophic hearts and highest relative dynamic thickening in normal patients. To partially compensate for the limits of considering a fully axisymmetric parameterization, we accounted for eventual rigid rotations and translations to better align parameterized and segmented ventricles throughout the cardiac cycle. This ensured us overall good fitting results, especially for the failing hearts, which proved to be more symmetric. Nonetheless, the methods here presented could be promptly extended also to non-axisymmetric parameterizations such as those based on non-uniform rational B-splines at the expense of extending the parameter space to additional dimensions.

Out of the several field views provided in the Sunnybrook database, we restricted our analyses to short-axis stack series, which have the disadvantage of providing relatively low resolution in the coronal planes. As a result, some artifacts were particularly evident close to the apex of the ventricle, where the segmentation and subsequent parameterization were sometimes not able to resolve correctly the apical thickness, especially in the thinner failing LVs. Not surprisingly then, the *H* parameter showed the largest relative standard deviations within the same cardiac cycle for all patients, indicating that apex parameterization accuracy could be likely corrected by registering and merging multiple MRI views.

### 4.2. Ventricular unloading

Standard FE simulations need to be initialized from an unloaded state, which cannot be directly extracted from images because ventricles are pressurized in all of the configurations imaged by cine-MRI or CT scans. Given material properties and inner LV pressure, iterative approaches such as the fixed point iteration method allow to estimate the unloaded configuration by progressively correcting a loaded state (Sellier, 2011; Genet et al., 2015). Nonetheless, due to their large computational cost and added complexity, these techniques are not typically incorporated into sophisticated optimization schemes proposed to estimate model parameters from images (Asner et al., 2016, 2017; Nasopoulou et al., 2017). To ensure feasibility, many modeling studies tend instead to use representative loaded configurations (i.e., at beginning or end of diastole) as approximations for the unknown unloaded state. As shown by our analyses, this could significantly bias results, since BoD and EoD configurations tend to match poorly to the profiles of unloaded geometries (see Figure 6). GP regression models of unloading can help circumvent some of the limitations associated with iterative methods and enable larger parameter search studies. Somewhat surprisingly, even a training set of *n*_train_ = 75 forward simulations was sufficient to ensure good inverse estimation results. LV profiles inferred from the statistical model matched those obtained via fixed point iteration with Dice scores always larger than 0.90 under 2 loading pressures and for 3 different sets of material properties. Considering that in our experience 7–10 iterations are needed to reach convergence via fixed point iteration, the preparation of an accurate statistical model might then require a computational cost comparable to unloading 7–10 ventricles with the standard method. Unlike fixed point iteration our strategy requires also an additional step of re-parameterizing simulation results in a format that can be handled by the machine learning model. The computational cost of reparameterizing is often negligible (on the order of few CPU mins), and after training the statistical model can be further interrogated to unload additional geometries at essentially no computational cost.

In addition to morphology, estimating the unloaded configuration relies on the knowledge of loading conditions and of the material properties of the myocardium. To fully characterize the material behavior of cardiac tissue, sophisticated experiments are required to reproduce *in vitro* the principal strain modes experienced by the heart during the cycle. The most extensive dataset on the passive behavior of the human myocardium is provided by Sommer et al. (2015). This work confirms how the micro-architecture of myocardial sheets leads to complex nolinear anisotropic behavior combined to a persisting viscoelastic response. Although viscoelastic effects were neglected in this work, we considered material properties based on the triaxial experiments of Sommer et al. (2015) as well as 2 other sets of constitutive behaviors based on experiments on animal models (Usyk et al., 2000; Wang et al., 2013; Gültekin et al., 2016). Our unloading procedure proved to work well for all of these sets of material properties.

In the form presented herein, our method for ventricular unloading required building a new training dataset and subsequently a new GP regression model for each set of material properties considered. Nonetheless, for future applications, the input parametric space could be extended to additional dimensions to account also for variations in material properties. While more training simulations would likely be needed to reach the desired convergence, the presented approach could still prove to be convenient for material property identification based on strain energy functions with reduced number of parameters (e.g., Nasopoulou et al., 2017), and especially in cases where large high performance machines are available to tackle the required computational cost in a distributed fashion.

The diastolic fiber strain at midwall, the constitutive law, and the shape of the ventricles at end-diastole are sufficient to uniquely unload geometries either via fixed-point iteration or GP regression. In this paper, we proposed to constrain end-diastolic fiber stretch to account for scenarios where diastolic pressure in the ventricles is not known. Animal model experiments suggest that end-diastolic fiber strain varies within a relatively small range in several circumstances (e.g., see Ross et al., 1971). Inspired by studies on inverse stress identification (Miller et al., 2010; Miller and Lu, 2013), we therefore tried to find the unloaded ventricular shape without solving for ventricular pressure. This was also motivated by the fact that unloading by strain would yield the same unloaded configuration independently from a homogeneous scaling of the constitutive law (i.e., predicted end-diastolic pressures would scale accordingly). To illustrate the potential of such approach, we additionally computed Dice scores between unloaded ventricles with 10% diastolic fiber strain using different constitutive laws. Our results (Dice scores of 0.90±0.05 for *W*_*U*_ vs. $W_{HO}^{G}$, 0.85±0.03 for *W*_*U*_ vs. $W_{HO}^{W}$ and 0.96±0.03 for $W_{HO}^{G}$ vs. $W_{HO}^{W}$, respectively) suggested strong similarity between unloaded ventricles endowed with umlaut Gultekin and Wang material behaviors, which followed the same Holzapfel-Ogden functional formulation.

### 4.3. Modeling of infarct mechanics

Two main factors increase the complexity of ischemia and myocardial infarction models. The first one is the need to account for the progressive changes in passive and active material properties that are triggered by the lesion and driven by tissue damage recovery and remodeling (Holmes et al., 2005). The second one is the more complex numerical framework required to handle the large finite element meshes needed to accurately capture realistic infarct shapes. In the past, only few studies have simulated non-transmural infarcts (Leong et al., 2015; Duchateau et al., 2016; Leong et al., 2017), while most models have either simulated infarct with simplified morphologies, or have allowed infarct/ischemic regions crossing the finite element boundaries (e.g., Mazhari et al., 2000; Jie et al., 2010; Wenk et al., 2011; Mojsejenko et al., 2015). Here, we present a model of non-transmural infarct that has refined elements in the border region of infarct. To handle large finite element meshes that result from such a refinement, we use an iterative solver for the large system of linearized equations with an efficient preconditioner (Gurev et al., 2015). To quickly summarize our results, the 2 main parameters affecting simulated SV were the transmural and circumferential extensions of the lesion, while location of the infarct played a minor role. Our models of infarct and the corresponding statistical model are still at a preliminary stage of development, and were here presented mainly to demonstrate the concept of integration between statistical and physical models.

### 4.4. Summary

This work shows 2 applications of GP regression in modeling ventricular heart mechanics. First, we present a strategy to estimate the ventricular unloaded configuration given material properties and intraventricular pressure (or alternatively fiber strain at midwall). Once an upfront computational cost (amounting to ~10 applications of a conventional iterative method) is paid for training, GP regression models allow the estimation of unlimited unloaded geometries at no additional cost. The method is therefore suitable to be used in analyses involving large number of patients such as those collected in publicly available databases. Second, we use GP regression as a convenient tool to explore results of a parametric study investigating coupled effects of infarct shape and location. While just a proof of concept study, these preliminary results demonstrate the power of the approach. That is, we were able to characterize a large variation in infarct location and size, including non-transmural infarcts with highly complex meshes that are computationally demanding to solve.

## Author contributions

PD designed the study, analyzed results, and wrote the manuscript, AH provided the image segmentations and supervised the image processing pipeline, SK performed the parametric infarct simulations, OS supervised the infarct simulations, JR wrote the manuscript and supervised the project; VG designed the study, wrote the manuscript, and supervised the project. All authors agree to be accountable for the content of the work.

### Conflict of interest statement

PD, AH, JR, and VG were employed by IBM Research. The remaining authors declare that the research was conducted in the absence of any commercial or financial relationships that could be construed as a potential conflict of interest.
