# Supplementary material for: Gaussian Process Regressions for Inverse Problems and Parameter Searches in Models of Ventricular Mechanics
Source: Front Physiol. 2018 Aug 14;9:1002. doi: 10.3389/fphys.2018.01002 (PMC6102646; doi:10.3389/fphys.2018.01002)
Supplement: Supplementary file 1 [file Data_Sheet_1.pdf]

# Gaussian Process regressions for inverse problems and parameter searches in models of ventricular mechanics

**P. Di Achille [1], A. Harouni [2], S. Khamzin [3,4], O. Solovyova [3,4], J.J. Rice [1], and V. Gurev [1,*]**

[1] *Healthcare and Life Sciences Research, IBM T.J. Watson Research Center, Yorktown Heights, NY, US*

[2] *IBM Research Almaden, San Jose, CA, USA*

[3] *Ural Federal University, Ekaterinburg, Russia*

[4] *Institute of Immunology and Physiology UB RAS, Ekaterinburg, Russia*

Correspondence*:
Viatcheslav Gurev, IBM T.J. Watson Research Center, 1101 Kitchawan Rd, Yorktown Heights NY 10598, US
vgurev@us.ibm.com

## SUPPLEMENTAL MATERIAL

### GP model of heartbeat kinematics

While the Sunnybrook Cardiac database consistently provides 20 frames per cardiac cycle for all scans, the metadata in the acquisition files revealed non-negligible variations in heart rate among patients. In order to quantify how such variations would affect cardiac phase durations for different categories of subjects, we developed an automatic procedure to de-noise segmentation and parameterization results and to robustly extract timings of cardiac phase changes. First, a GP regression was fitted to the time evolutionof the 6 geometric parameters describing LV geometry over the cardiac cycle. For the GP, we assumed RBF kernels with unitary a priori variance, and then used the open-source package GPy to perform an a posteriori optimization (GPy, 2018). Mean value estimates of the fitted process provided a smoothed and differentiable representation of the geometric parameter evolution. To split the cardiac cycle among its various phases, we carefully examined image datasets for each patient and recorded the frame numbers at which the aortic and mitral valves visibly opened and closed.

Analysis of LV volume time series with GP regression provided us with an objective strategy to split the time course of MRI frames among the different cardiac phases. Figure 1A shows volume changes over time for the different patient categories, both in a raw form (i.e., computed directly from best-fit 6-parameter models, first row) and after de-noising with GP regression (i.e., computed as mean expected value of the process, second row). Volume behavior recapitulated globally what already observed in the 6-parameter analysis such as the low contractile performance of HF patients. In addition, the volume analysis made clear some striking difference between categories of patients. For example, there is much greater variability in the LV volume in the HF cases (compare HF-NI and HF-I to the N and HYP traces in the first row of Figure 1A). The second row also shows average cardiac phase splits (see dashed lines). Absolute time values corresponding to each phase were assigned after extracting heart rate information from the DICOM metadata. Average values of these timings are plotted in Figure 1B and reported in Table 1.

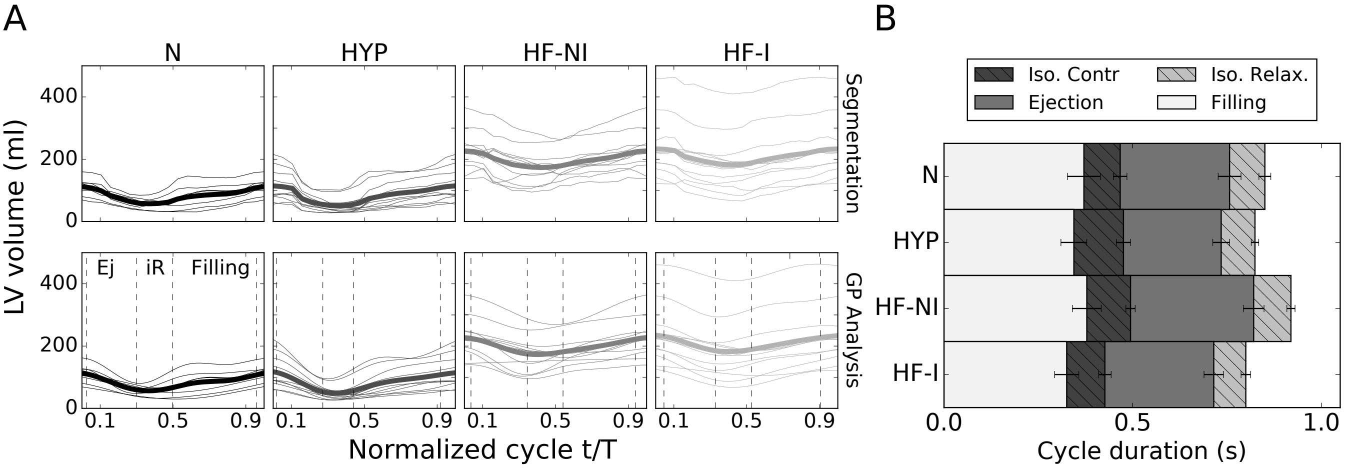


**Figure 1.** Estimation of cardiac phase duration via GP fitting and analysis. A) Volume changes over a normalized cardiac cycle are shown un-processed (see first row) and after GP analysis (see second row) for the 4 groups (N=normal subjects, HYP=hypertrophic patients, HF-NI=heart failure patients, HF-I=heart failure patients with infarct). Traces for each patients are shown as thin line, and group averages as thick lines. Also shown are subdivisions of the cardiac cycle into phases averaged over each group of patients (vertical dashed lines). Ej = ejection phase, iR = isovolumetric relaxation, Filling = filling phase. B) Barplot of group averages of cardiac phase durations. Error bars indicate standard error of the mean for each group.

## Detailed infarct model

The varying infarct shapes necessary to build a GP regression model of infarct contraction were generated by a custom meshing procedure operating on a baseline geometry from the database (I-02). Outcomes of the protocol were high resolution meshes in which infarcts chosen via latin hypercube sampling according to the parameterization scheme introduced in section 2.6 were accurately discretized maintaining internal element faces aligned along their lesion boundaries. The border region surrounding the infarct was also

**Table 1.** Average heart rates and cardiac phase durations extracted from the Sunnybrook database

| | HR (bpm) | Iso. Contr. (s) | Ejection (s) | Iso. Relax. (s) | Filling (s) |
|---|---|---|---|---|---|
| N-02 | 62 | 0.097 | 0.340 | 0.048 | 0.480 |
| N-03 | 56 | 0.054 | 0.380 | 0.054 | 0.590 |
| N-05 | 73 | 0.082 | 0.370 | 0.041 | 0.330 |
| N-06 | 74 | 0.081 | 0.280 | 0.160 | 0.280 |
| N-07 | 59 | 0.150 | 0.360 | 0.150 | 0.360 |
| N-09 | 57 | 0.160 | 0.310 | 0.052 | 0.520 |
| N-10 | 63 | 0.190 | 0.290 | 0.140 | 0.330 |
| N-11 | 67 | 0.090 | 0.270 | 0.130 | 0.400 |
| N-40 | 92 | 0.065 | 0.260 | 0.097 | 0.230 |
| HYP-01 | 64 | 0.047 | 0.330 | 0.047 | 0.520 |
| HYP-03 | 72 | 0.250 | 0.250 | 0.042 | 0.290 |
| HYP-06 | 88 | 0.140 | 0.240 | 0.140 | 0.170 |
| HYP-07 | 70 | 0.085 | 0.260 | 0.130 | 0.380 |
| HYP-08 | 53 | 0.110 | 0.400 | 0.110 | 0.510 |
| HYP-09 | 69 | 0.087 | 0.310 | 0.130 | 0.350 |
| HYP-10 | 59 | 0.250 | 0.250 | 0.051 | 0.460 |
| HYP-11 | 70 | 0.210 | 0.260 | 0.085 | 0.300 |
| HYP-12 | 61 | 0.150 | 0.300 | 0.098 | 0.440 |
| HYP-37 | 68 | 0.130 | 0.310 | 0.088 | 0.350 |
| HYP-38 | 91 | 0.099 | 0.200 | 0.099 | 0.260 |
| HYP-40 | 81 | 0.074 | 0.220 | 0.110 | 0.330 |
| NI-03 | 53 | 0.110 | 0.450 | 0.110 | 0.450 |
| NI-04 | 51 | 0.059 | 0.410 | 0.120 | 0.590 |
| NI-07 | 80 | 0.190 | 0.260 | 0.150 | 0.150 |
| NI-11 | 71 | 0.130 | 0.340 | 0.042 | 0.340 |
| NI-12 | 63 | 0.140 | 0.430 | 0.095 | 0.290 |
| NI-13 | 72 | 0.120 | 0.290 | 0.083 | 0.330 |
| NI-14 | 49 | 0.190 | 0.370 | 0.120 | 0.560 |
| NI-15 | 59 | 0.150 | 0.360 | 0.051 | 0.460 |
| NI-31 | 57 | 0.110 | 0.370 | 0.160 | 0.420 |
| NI-33 | 66 | 0.091 | 0.270 | 0.091 | 0.450 |
| NI-34 | 65 | 0.092 | 0.320 | 0.092 | 0.410 |
| NI-36 | 69 | 0.087 | 0.300 | 0.130 | 0.350 |
| I-01 | 78 | 0.077 | 0.310 | 0.077 | 0.310 |
| I-02 | 80 | 0.150 | 0.220 | 0.110 | 0.260 |
| I-04 | 65 | 0.180 | 0.370 | 0.046 | 0.320 |
| I-05 | 59 | 0.100 | 0.200 | 0.150 | 0.560 |
| I-06 | 88 | 0.034 | 0.200 | 0.170 | 0.270 |
| I-07 | 53 | 0.230 | 0.400 | 0.057 | 0.450 |
| I-08 | 78 | 0.120 | 0.310 | 0.120 | 0.230 |
| I-09 | 90 | 0.067 | 0.300 | 0.033 | 0.270 |
| I-10 | 75 | 0.080 | 0.280 | 0.040 | 0.400 |
| I-11 | 72 | 0.083 | 0.330 | 0.083 | 0.330 |
| I-12 | 62 | 0.048 | 0.440 | 0.048 | 0.440 |
| I-40 | 73 | 0.120 | 0.290 | 0.120 | 0.290 |
| N_avg | 61 ± 21 | 0.102 ± 0.048 | 0.291 ± 0.0962 | 0.092 ± 0.049 | 0.364 ± 0.140 |
| HYP_avg | 66 ± 20 | 0.130 ± 0.068 | 0.260 ± 0.081 | 0.089 ± 0.036 | 0.340 ± 0.124 |
| NI_avg | 59 ± 17 | 0.116 ± 0.045 | 0.326 ± 0.099 | 0.099 ± 0.039 | 0.378 ± 0.138 |
| I_avg | 68 ± 20 | 0.104 ± 0.056 | 0.287 ± 0.096 | 0.085 ± 0.0452 | 0.325 ± 0.116 |

meshed at high resolution, an important feature for future studies aiming to characterize the mechanical environment following infarction.

Input required by the procedure was a standard LV tetrahedral mesh used as baseline for infarct insertion. The 3 parameters prescribing infarct location and surface extension (i.e., Long., $\Delta\mathrm{Long.}$ and $\Delta\theta$, see section 2.6) were sufficient to label a subset of endocardial element faces as infarcted. Subsequently, a distance field ($\phi$) was computed by solving the weak form of the eikonal equation via finite elements,

following an approach similar to Young and Panfilov (2010). A zero-distance boundary condition was applied to the infarct-labeled faces, and the resulting distance field was then normalized via linear scaling to have a unitary value at the epicardial nodes closer to the infarct. The transmural boundary of the infarct was then extracted as an isosurface of the normalized distance field matching the value of the Depth parameter (see section 2.6). Similarly, the border zone boundary was extracted as the distance isosurface corresponding to 2 times the Depth value. We finally combined the capabilities of the ACVD surface refiner (Valette et al., 2008) and of the Tetgen tetrahedral mesh generator (Si, 2015) to create meshes that conserved the internal boundaries of the infarct and of the border zone. Tetgen options were employed to impose arbitrarily high accuracy in the border region (see Figure 2).

Values for the parameters of the model by Lumens et al. (2009) used to drive active contraction of the myofilament are reported in Table 2.

**Table 2.** Lumens model parameters

| Parameter | Value |
|---|---|
| BCL | 600 |
| $C_{\text{rest}}$ | 0.0 |
| $L_{s0}$ | 1.51 |
| $L_{\text{serel}}$ | 0.04 |
| $v_{\max}$ | 0.007 |
| $\tau_d$ | 32 |
| $\tau_r$ | 48 |
| $\tau_{\text{sc}}$ | 425 |
| $\sigma_{\text{act}}$ | 120 |

### Dice score for geometry similarity

To avoid possible errors introduced by projecting actual LV shapes to the idealized geometry space, comparisons between unloading algorithms and imaged diastolic configurations (i.e., BoD, EoD, and OptD) were carried out on geometries discretized following the same algorithm used for the biomechanical simulations. After volumetric meshing, triangulated cross-sections were extracted along the longitudinal plane via custom VTK routines. Subsequently, elements from the first cross-section (A) whose centers were closer than 0.01 mm to the second cross-section (B) were considered as intersecting (i.e., $I_{AB}$). To make the metric commutative, we similarly labeled as intersecting also the centers of the second cross section closer than 0.01 mm to the first one (i.e., $I_{BA}$) and finally computed the Dice score

$$\text{Dice} = \frac{I_{AB} + I_{BA}}{A + B} \tag{1}$$

where $I_{AB} + I_{BA}$ is the sum of the areas of all of the intersecting elements, and $A + B$ is the sum of the areas of the two cross-sections. Note how full intersection leads to a Dice score of 1.0.

## REFERENCES

[Dataset] (2018). GPy: Gaussian processes framework in python

Lumens, J., Delhaas, T., Kirn, B., and Arts, T. (2009). Three-Wall Segment (TriSeg) Model Describing Mechanics and Hemodynamics of Ventricular Interaction. *Annals of Biomedical Engineering* 37, 2234–2255. doi:10.1007/s10439-009-9774-2

Si, H. (2015). TetGen, a Delaunay-Based Quality Tetrahedral Mesh Generator. *ACM Trans. Math. Softw.* 41, 11:1–11:36. doi:10.1145/2629697

Valette, S., Chassery, J. M., and Prost, R. (2008). Generic remeshing of 3D triangular meshes with metric-dependent discrete Voronoi diagrams. *IEEE Transactions on Visualization and Computer Graphics* 14, 369–381

Young, R. J. and Panfilov, A. V. (2010). Anisotropy of wave propagation in the heart can be modeled by a Riemannian electrophysiological metric. *Proceedings of the National Academy of Sciences* 107, 15063–15068
